# Effects of Rotational Motion in Robotic Needle Insertion

**Published:** 2015-12-01

**Authors:** H. Ramezanpour, H. Yousefi, M. Rezaei, M. Rostami

**Affiliations:** 1 Department of Mechanical Engineering, Iran University of Science and Technology, Iran; 2 Auckland Bioengineering Institute, University of Auckland, New Zealand; 3 Department of Electrical Engineering & Mechatronics, Islamic Azad University, Iran; 4 Department of Biomedical Engineering, Amirkabir University of Technology, Tehran, Iran

**Keywords:** Force-Displacement Diagram, Friction, Soft Tissue Insertion, Rotational Capability

## Abstract

**Background:**

Robotic needle insertion in biological tissues has been known as one the most applicable procedures in sampling, robotic injection and different medical therapies and operations.

**Objective:**

In this paper, we would like to investigate the effects of angular velocity in soft tissue insertion procedure by considering force-displacement diagram. Non-homogenous camel liver can be exploited as a tissue sample under standard compression test with Zwick/Roell device employing 1-D axial load-cell.

**Methods:**

Effects of rotational motion were studied by running needle insertion experiments in 5, 50 and 200 mm/min in two types of with or without rotational velocity of 50, 150 and 300 rpm. On further steps with deeper penetrations, friction force of the insertion procedure in needle shaft was acquired by a definite thickness of the tissue.

**Results:**

Designed mechanism of fixture for providing different frequencies of rotational motion is available in this work. Results for comparison of different force graphs were also provided.

**Conclusion:**

Derived force-displacement graphs showed a significant difference between two procedures; however, tissue bleeding and disorganized micro-structure would be among unavoidable results.

## Introduction

Most common minimal invasive surgery procedures in medicine for radioactive seed plantation, injection and many other aspiration methods are currently using needle insertion for their sampling procedures and therapies[1]. Although it is not the only available method for direct reaching to intended targets in human body through biological soft tissues, it showed more efficient results during testing than other ways in minimal invasive surgery. In all robotic needle steering procedures, needle faces a highly soft and mostly non-homogenous tissue, while this situation makes it difficult to reach targets accurately[2-5]. These problems exacerbates on having an efficient accuracy when facing ex-vivo testing condition[1, 3]. Therefore, the probability of finding an accurate trajectory on insertion remains hapless[6]. Through minimum requirement of real-time factor in the process of surgical simulation tools, intra-operative image registration on surgical procedures must not be interrupted by the work flow. Many applications of percutaneous needle insertion are observed in different works as biopsies, brachytherapy and neurosurgery[7-10]. Other applications of needle insertion such as deep needle insertion have been used in the failure mechanism of ventricular tissue and some phenomenological models derived based on general needle steering experiments. These models have been developed to describe the most efficient collection of terms in force-displacement graph of insertion. Through acquiring data from biological tissues and developing models appropriate for application in simulation for the applications of robot-assisted surgery or impedance control of MRI compatible robots, there are some complexities and difficulties due to tissue deformation, non-homogeneity and opacity as well as many possible sources of forces applied to surgical tools including probe, needle and catheter[11]. Also, having real-time computational factors such as accurate modelling of biological soft tissue mechanical properties are among minimum requirements of systems for surgical simulations[12]. In addition, there is no defined tolerance for the accuracy of needle insertion in clinical trials and all efforts in surgeries by physicians observed on obtaining a less misplacement in the effectiveness of the needle insertion[13, 14]. In this significantly non-accurate condition, some extra errors might be included due to manual needle insertion operation of physicians and clinical trainers. Through all these, modelling of forces during needle insertion into biological soft tissue is an important aspect of any study for accurate surgical simulation[1]. In this regard, force modelling of soft tissue insertion has been an important challenge during recent years. The available force models developed in the literature might be used in surgical simulation and robot-assisted surgeries[1-3]. Accurate models are not only important for haptic feedback, but they also determine the way tissue deforms during contact with a surgical tool. Through these procedures, some complications have arisen and most of them are due to imprecise placement of surgical instruments. This is mostly because of unequal dosage distribution or damage on delicate structures that can be involved by the structure of the tissue at surgery site. Herein, the identification of tissue characteristic for pre-operative surgeries is a challenge in future works. In this work, a short history of force modelling and numerical simulation of needle insertion into biological soft tissue is discussed. Then, in order to understand the effects of rotational motion and angular velocity in needle insertion force graph, many number of tests in different directional velocities and angular velocities were enacted on soft biological tissues. Finally, some experiments prompted to show the amount of friction forces in the process of insertion.

### Needle Insertion Procedure

Everything affecting the process of insertion of a needle into soft tissue must be considered in this part; however, as most of related articles discussed, these phases might be decomposed into two completely different phases for constant velocity of needle soft tissue interaction (quasi-static insertion)[1-3,5, 15]. As we will state in what follows, the first two stages of the whole procedure are from a different nature.

#### Phase 1: Non-Linear Elastic Deformation

The needle tip comes into contact with the tissue and deforms it without any penetration. It continues with a small puncture at the end of the first phase

#### Phase 2: Steady-State Visco-Plastic Penetration

Once an energy threshold is reached, the liver capsule ruptures and a crack initiates. Then, steady state penetration initiates and the force increases with depth. This phase finishes when the needle tip stops at target point. In the literature many of the authors discussed two more phases for continuing the process of sampling with tissue relaxation and also extraction or so-called withdrawal phase as final phases of the insertion procedure[1-3,5].

### History of Needle Insertion Testing

Most experimental works in this field present a robotic needle insertion with a non-human soft tissue or a phantom tissue, each of which provides this ability to opt the stiffness coefficient of testing sample[2, 3]. It can be noted that these tissues are all non-homogeneous and using homogeneous tissues for experiments is different from reality. The majority of experimental works have used constant velocities which might be a doubt for replacement by constant force. These tests are quasi-static and have an advantage of eliminating inertia term in force model[3, 15]. Although in most experiments a special geometry was cut, exploiting a fixture prevented the threshold of buckling for phantom tissue on Dimaio’s work[15]. In some other works, authors used an expression of fast needle insertion. In this phenomenon, the idea is to define a gauge for amount of pain. Near 50% difference in force-displacement diagram for one hundred times variation in needle tip velocity was presented[5]. However, this is never related to tissue deformation when velocity of insertion differs. In some works, authors do not make use of any types of fixture for their test and they used larger amounts of tissue for needle insertion. These conditions provide more real data for the procedure; however, they make the mathematical modelling a little harder to verify. More complexities appear while we are approaching to have samples closer to human soft tissue. Obviously, researchers define easier style of the tests in order to have a simpler assumption in formulating the procedure[1]. Other methods were used to determine the amount of friction force between the needle shaft and boundaries of ruptured area inside tissue.

In the categorization of different types of involving forces, friction force can be measured and modelled easier by moving the inserted needle with a sinusoidal function inside a tissue to apply a Karnop model[3]. This method which was implemented by Okamura claims that describing the friction model is significantly complex while for 4 frequent frequencies of needle motion, behavior of the tissue indicates very small variation between them[3]. This method was established on the basis of hysteresis theory. Another type of experiment used to determine the amount of friction force was used with deep penetration in the definite thickness of the tissue with constant velocity[1].

## Materials and Methods

### Needle Insertion Formulation

As a simple approach, the whole system is simulated by a linear set of mass, spring and damper in which the mass represents the inertial force and needle acceleration coefficient, damper relates to the velocity coefficient and spring takes the role of displacement coefficient[2]. Later, we should switch from elastic phase to plastic since the process of tearing the tissue by needle is not fully elastic and after certain amount of needle displacement, tearing occurs. Moreover, friction happens at needle-tissue boundary and it is linearly related to needle diameter and insertion depth. In the presence of elastic phase, this friction makes a phenomenon known as stick-slip happening which can be studied more in further studies. Therefore, force-displacement equation could be stated as follows[1, 13]:

$$F_{z}=Kz+b\dot{z}+m\ddot{z}+f_{f}.sgn\left(\dot{z}\right)+k{<z-z_{0}>}^{1}$$

In this formulation, the last term presents the first order Macouli parenthesis. This formulation can be considered as a comprehensive model in force modelling of needle insertion. However, by considering a quasi-static approach and eliminating the inertia terms, more simple models can be achieved. Other factors such as rotational velocities were not considered in this model. 

### Testing Conditions

**Figure 1:**
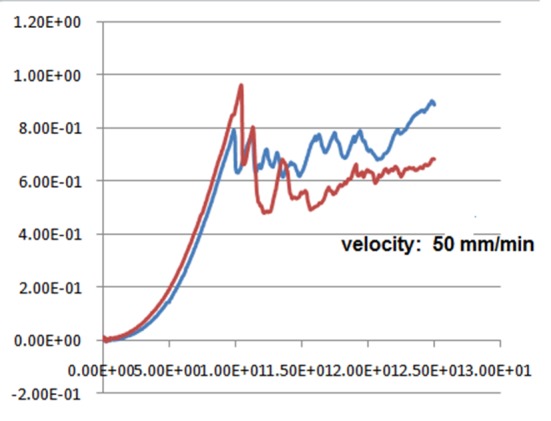
Repeatability of experiments and appearance of two different phases on needle insertion into soft biological tissue

Experimental results are derived from the insertion of a 8.5 centimetre length, 1.54mm diameter, 220 bevel tip into a non-homogeneous camel liver with directional velocities of 5, 50, 200 mm/min.  The angel of insertion was perpendicular to the surface of the tissue. In all these tests, soft tissues were cut with dimensions of 5*5*3cm^3^ and no restriction was applied on top side. While it always enjoys totally in the mechanism of testing from soft biological tissues, it is better to use the original condition of the human body. For some special applications, it might be needed to fix tissue for testing. Generally, for the whole mechanism of needle insertion, two different phases occur in this process, in which subsequent document and some related articles were observed. As it was observed in Figure 1 the same phase of insertion in this figure is obvious and thus the regional maximum force is in points when small puncture occurs during the penetration in tissue surface.

### Using Zwick/Roell for Experiments

**Figure 2:**
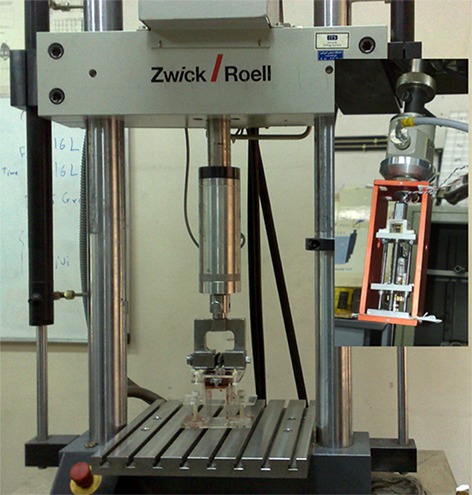
Completing experiments with Zwick/Roell desktop  in order to obtain force-displacement diagram in different axial velocities

In order to obtain the amount of force in axial direction of needle motion in the mechanism, an accurate load-cell with Zwick/Roell desktop which is shown in Figure 2 to sense the opposed force of axial loading direction was introduced. We exploited a Zwick/Roell desktop mark number HCT/405/25 toolkit fabricated in 1998, having the accuracy of 0.01 N and 0.001 mm axial force resolution. In order to have better experimental samples, the system enjoyed a 20 N load cell with 1000 discretizing points.

On the other hand, high porosity rate of camel liver was used to simulate the condition of human body tissue. There are two main reasons behind this decision. One of them is the tissue will be affected by the transition of time in in-vivo test rather than ex-vivo which might better describe and simulate the condition of body and because of its porous material, camel liver is talented to hold blood in the pores. Secondly, there exists a similarity between camel liver and human liver tissue[1].

### Using Rotational Mechanism and Camel Liver as Materials

**Figure 3:**
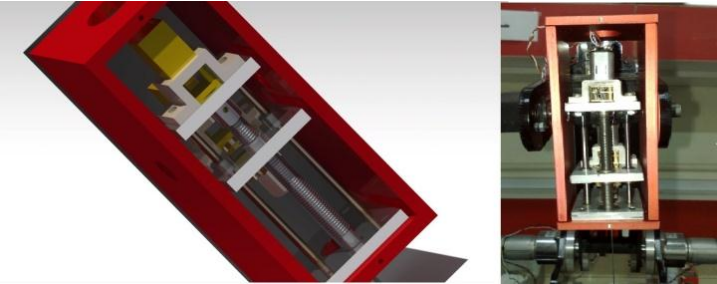
Designed mechanism of supporting rotational degree of freedom and capability of giving angular velocity

In order to compare the effects of rotational velocity in needle insertion, some experiments supported to bring the advantages and disadvantages of rotational velocity in the process of needle insertion into soft biological tissues. For this purpose a manipulator similar to Figure 3 was required.

In trajectory planning for the needle inside tissue, most parameters are related firstly to reaching target without hitting polygonal obstacles and secondly to decrease the amount of force and damage on ruptured tissue. While with rotation, the probability of increment in distortion energy appears and it might be considered that this amount of energy would be the next criteria for the modelling of damage in soft tissue, the first one as mentioned in some related articles was the displacement of nodes in finite element meshing. In these applications, it must be added that through the ability of rotational mechanism, physicians can guide needle in a definite direction but it might be assembled by the amount of velocity to obtain favourable displacement in that direction, and then change into new path by another amount of velocity. Other notions that might be useful are the efficiency of the needle material and the tissue characteristic; thinner needles are more flexible and thus result in less pressure and damage to the tissue. They allow making curved trajectories and accomplishing easier obstacle avoidance. They are, however, very difficult to control. On the other hand, flexible needle navigation inside the tissue is very complicated and requires implementing image processing abilities.

**Figure 4:**
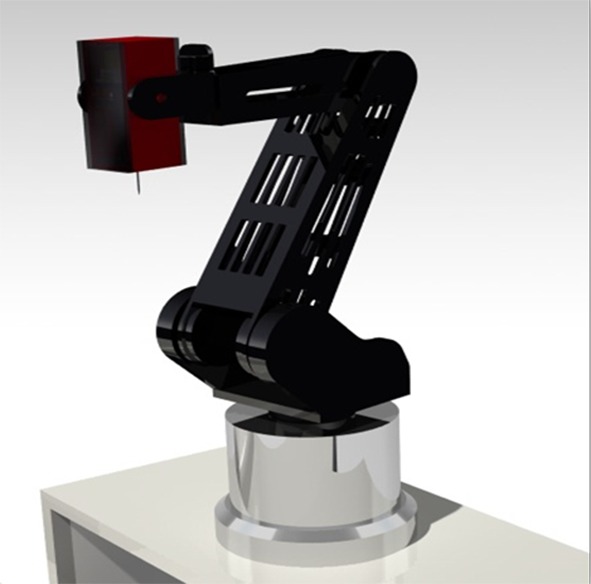
Body of RAMA Robot

Here some numbers of needle insertion tests with angular velocities of 50 rpm, 100 rpm, 150 rpm and 300 rpm gathered. Force-displacement graph for these tests are involved in a table with the condition of the same fixture and the same device for non-rotational form. So, there are some directional constant velocities in these procedures, both in general insertion and in with angular velocity insertion. Another application of this mechanism is presented on radio-injection RAMA robot in Figure 4.

### Friction Force Tests

In order to calculate the amount of friction force included in total force of penetration in the process of needle insertion, there could be some methods which might come useful. As it discussed before, by using a deep penetration for a definite thickness of a soft biological tissue in the same material and similar working condition, we can have a good understanding of the friction force. This work aims to discover the effect of friction in total force during needle insertion procedure. In this section, three different velocities were tested to examine the amount of affecting friction force. For all experiments, tissue thickness of nearly 20 mm was considered.

**Figure 5:**
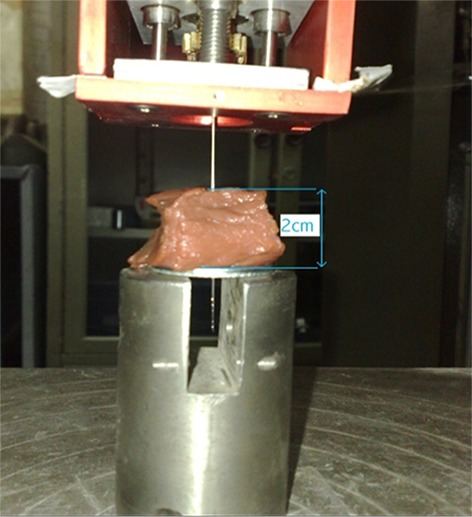
Deep needle insertion to definite tissue thickness in order to measure the amount of friction force

Absolute friction force is dependent on the amount of needle shaft interacted and in contact with soft tissue ruptured area. In these types of tests, the amount of force after complete penetration to the tissue is observed to converge to a constant force for different directional velocity. In Figure 5, experimental set up for friction test is declared.

## Results and Discussion

**Figure 6:**
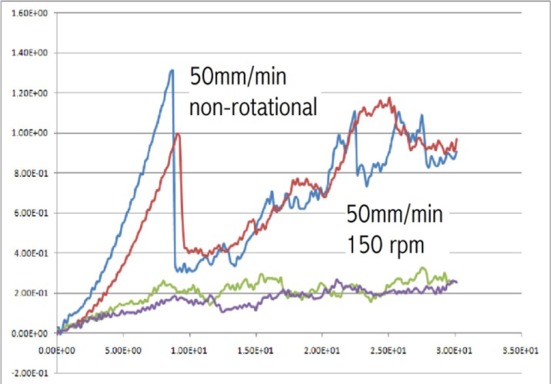
Compromise between two different needle insertions into soft tissue, with or without rotation with repeatability

In this part, the amount of forces for each penetration is within limited areas. Here the data presented in the table might show the compromise with two different results. As the results show, the magnitude of force decreases in the same directional velocities with the condition of rotation mechanism than the case of non-rotating needle force. Also, if the amount of friction degraded from the total magnitude of the needle force in non-rotating case, there are still more differences between two cases. It might be the effect of correlation in amount of force in needle insertion by new parameters involved in the process as angular velocity or so. As results indicate, there could be some more reasons for less effect of friction in the rotational case because of angular velocity and fast location of needle during direct penetration. These effects are more obvious than fast needle insertion characteristics and its related works. Another big difference between these two graphs shows the disappearance of the first phase of needle insertion force into soft biological tissues that might be because of the sharp edges of the needle. Here, in Figure 6 two phases of insertion for the needle penetration without insertion are observed and additionally the effect of rotation in decreasing the maximum force and disappearance of the punctuation point are witnessed.

**Figure 7:**
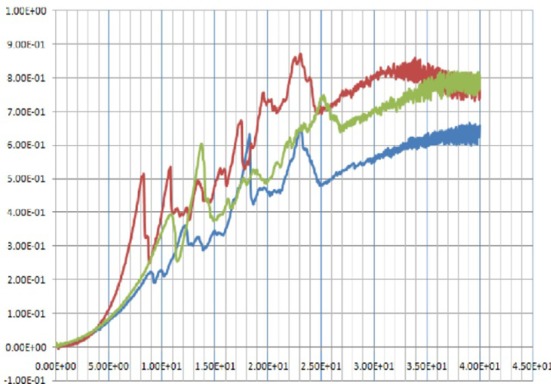
Friction force testing by the 3 different positions in order to see repeatability in magnitude of friction for definite tissue thickness in 50mm/min velocity

Provided results in Figure 7 for various tests of friction force show much obvious differences on its magnitude of by changing the applied velocities on manipulator. As it discussed in literature review, authors used this phenomenon to formulate the friction components of insertion force, however; it seems to be more complex than to be explained by a sample formula.

**Figure 8:**
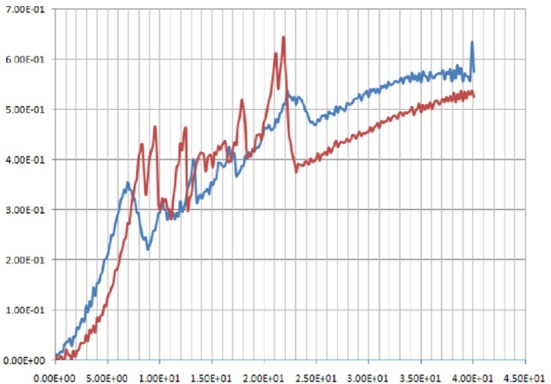
Friction force testing by the 2 different positions in order to see repeatability in magnitude of friction for definite tissue thickness in 200mm/min velocity

Figure 7 shows differences among 3 needle insertions into a constant thickness of the tissue in order to measure the amount of friction after totally penetrated to tissue. The amount of friction for different insertions varies between 0.6 N to 0.8 N for 20 mm thickness of the tissue in the velocity of 50 mm/min, which can be considered in Figure 8 as the experiment for repeatability.

The amount of friction for different insertions varies between 0.53 N to 0.64 N for 20 mm thickness of the tissue in the velocity of 200 mm/min. An important notion is that the amount of friction force does not vary linearly with the increment in velocity. Despite many studies such as Okamura[3], it is not just a function of velocity, and it might be correlated by other parameters involved in the insertion procedure.

**Figure 9:**
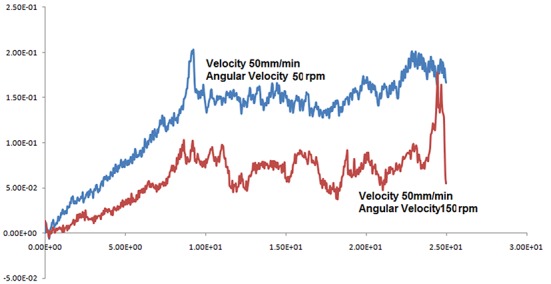
Compromise of needle insertion force with rotation during two different angular velocities for penetration

Figure 9 compares two different angular velocities emphasizing the increment in the amount of angular velocity on force of insertion. Again, at the first phase of the insertion, it disappears in the insertion with rotation. In this figure, between two different angular velocity of 50 and 150 rpm with directional velocity of the 50 mm/min, both data sets are acquired. We would better think about the correlation effects on force of needle insertion into soft biological tissues which is due to the change of angular velocity.

## Conclusion and Future Work

With respect to experimental results, needle might be in a tissue fully-inserted for many seconds. The behaviour of a tissue in vicinity of needle is similar to a plastic motion and in subsequent works. Visco-plastic model will be developed and also there might be some problems and disadvantages of the motion with rotation in case bleeding in liver or other related tissues. These might be the disadvantages of rotational process and it would be seen in in-vivo tests. Although it has not been seen in ex-vivo tests process for the behaviour of a tissue, after consulting with some physicians, the problem bleeding is such big that before the insertion of needle ability to eliminate the cancer resources is to be investigated. In this case, integrity of the surface density in the tissue can lead to the coefficient of plastic phase, and other things are about the initiation of using force control as the sensed information during the loop of controlling in the robot. In the future work, application of an impedance control for using the force control of the robot might be considered to gain feedback for sensate the magnitude of interaction in the needle shaft react at the back of the needle which jointed in the manipulator of the robot. In addition, in some related works the effect of new method of needle insertion (fast needle insertion instead of general low-speed insertion) for MR-guided, prostate implant robotic system has been observed. This work will continue with compromising the effects of needle tapping and it might have efficiency for the purpose of buckling investigation that might be explained in subsequent works like the effect of impulse instead of steady state penetration. 
